# In situ SU-8 silver nanocomposites

**DOI:** 10.3762/bjnano.6.168

**Published:** 2015-07-30

**Authors:** Søren V Fischer, Basil Uthuppu, Mogens H Jakobsen

**Affiliations:** 1 DTU Nanotech, Technical University of Denmark, Anker Engelundsvej 1, 2800 Kgs. Lyngby, Denmark

**Keywords:** functional photoresist, in situ synthesis, metal nanoparticles, micro and nanofabrication, nanocomposite

## Abstract

Nanocomposite materials containing metal nanoparticles are of considerable interest in photonics and optoelectronics applications. However, device fabrication of such materials always encounters the challenge of incorporation of preformed nanoparticles into photoresist materials. As a solution to this problem, an easy new method of fabricating silver nanocomposites by an in situ reduction of precursors within the epoxy-based photoresist SU-8 has been developed. AgNO_3_ dissolved in acetonitrile and mixed with the epoxy-based photoresist SU-8 forms silver nanoparticles primarily during the pre- and post-exposure soft bake steps at 95 °C. A further high-temperature treatment at 300 °C resulted in the formation of densely homogeneously distributed silver nanoparticles in the photoresist matrix. No particle growth or agglomeration of nanoparticles is observed at this point. The reported new in situ silver nanocomposite materials can be spin coated as homogeneous thin films and structured by using UV lithography. A resolution of 5 µm is achieved in the lithographic process. The UV exposure time is found to be independent of the nanoparticle concentration. The fabricated silver nanocomposites exhibit high plasmonic responses suitable for the development of new optoelectronic and optical sensing devices.

## Findings

Noble metal nanoparticles (NPs) have been of high interest for many years as their unique properties make them useable in a large variety of applications [1]. The application of these NPs ranges from optical imaging, optoelectronics and electrochemistry to catalysts [2]. However, it is difficult to use such NPs in conjunction with standard top down micro- and nanofabrication processes as positioning and control of the nanoparticles are impossible to maintain [3]. Homogeneous polymeric thin film metal nanocomposites are therefore of great interest within micro- and nanofabrication [4–6]. The nanoparticles encased in a polymeric matrix should maintain their physical properties, while the nanocomposite can be structured by using standard fabrication methods allowing for the development of new optoelectronic and sensing devices.

Metal nanoparticles in photoresists are also interesting within technologies for three-dimensional structuring as these can be used for fabrication of photonic crystals [7].

A good candidate for a polymeric matrix is the epoxy-based photoresist SU-8, which is widely used for making high-aspect-ratio structures [8]. SU-8 is good for optical sensors being highly transparent in the visible region [9] and also useful in biological sensing applications being quite biocompatible [10]. SU-8 is also well suited for direct laser writing and 3D structuring [11] although only 2D structures are considered in this work.

SU-8 thin films are deposited on wafers by using standard spin coating techniques [12]. However, high loadings of preformed NPs in the polymer change the rheological behaviour and might hinder the ability to use spin-coating for thin film nanocomposites [13–14]. Furthermore, it is difficult to obtain stable suspensions of preformed NPs in SU-8 without aggregation and phase separation.

In situ synthesis methods where the particles are formed directly within the polymeric matrix from a precursor can circumvent this problem. Here, we report a fast and simple method for fabricating homogeneous SU-8-based metal nanocomposite thin films with in situ generated silver nanoparticles. These composite materials can be deposited on wafers by using standard spin coating techniques and subsequently structured with UV lithography.

The nanocomposite is prepared by dissolving AgNO_3_ precursor in acetonitrile in a two-fold dilution series: 500 mg·mL^−1^, 250 mg·mL^−1^, 125 mg·mL^−1^, 62.5 mg·mL^−1^, 31.3 mg·mL^−1^, and 15.6 mg·mL^−1^. 0.5 mL of the freshly prepared precursor solutions or 0.5 mL acetonitrile as reference is added to 4 mL of SU-8 2002, which is a formulation of SU-8 with cyclopentanone as the main solvent. As AgNO_3_ is not soluble in cyclopentanone, acetonitrile is chosen as a co-solvent. AgNO_3_ precursor solutions with a concentration above 500 mg·mL^−1^ are immiscible with SU-8 using the described protocol.

The SU-8 mixture is then spun on a 100 mm fused silica or silicon wafer, at 1500 rpm for 1 min followed by heating at 95 °C on a hotplate for 10 min. After heating, a UV exposure of 7.5 min is performed to cross-link the polymer followed by a post exposure bake at 95 °C on a hotplate for 10 min. In case of structuring, a mask is used in soft-contact mode during the exposure and the wafer is then developed for 2 min in propylene glycol monomethyl ether acetate (PGMEA) [15] followed by rinsing with 2-propanol (IPA). After development or post exposure bake some of the wafers are further heated to 300 °C for 30 min on a hotplate. The UV exposure is done without any filters in the aligner.

The chosen co-solvent for the AgNO_3_ precursor, acetonitrile, is a mild reducing agent. This precursor solution must therefore be prepared fresh, and added to the SU-8 just before spin coating, to minimise unwanted formation of nanoparticles [13].

**Figure 1:**
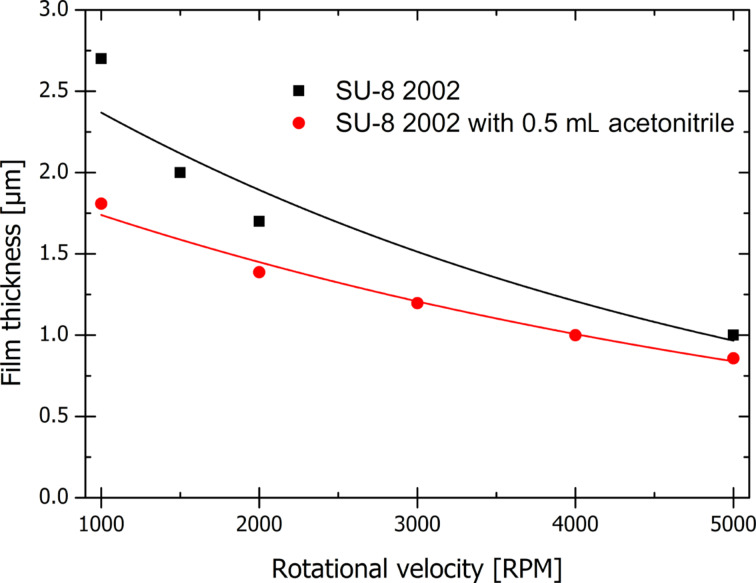
Spin curves for SU-8 2002 and SU-8 2002 with acetonitrile. The addition of acetonitrile results in a decreased film thickness compared to the unmodified SU-8.

The used formulation of SU-8 can be used for depositing thin films in the range of 1–1.8 µm as documented by the spin curve shown in Figure 1. The resulting film thickness is smaller than with unmodified SU-8, but the thickness can be increased by using more viscous SU-8 formulations if desired.

**Figure 2:**
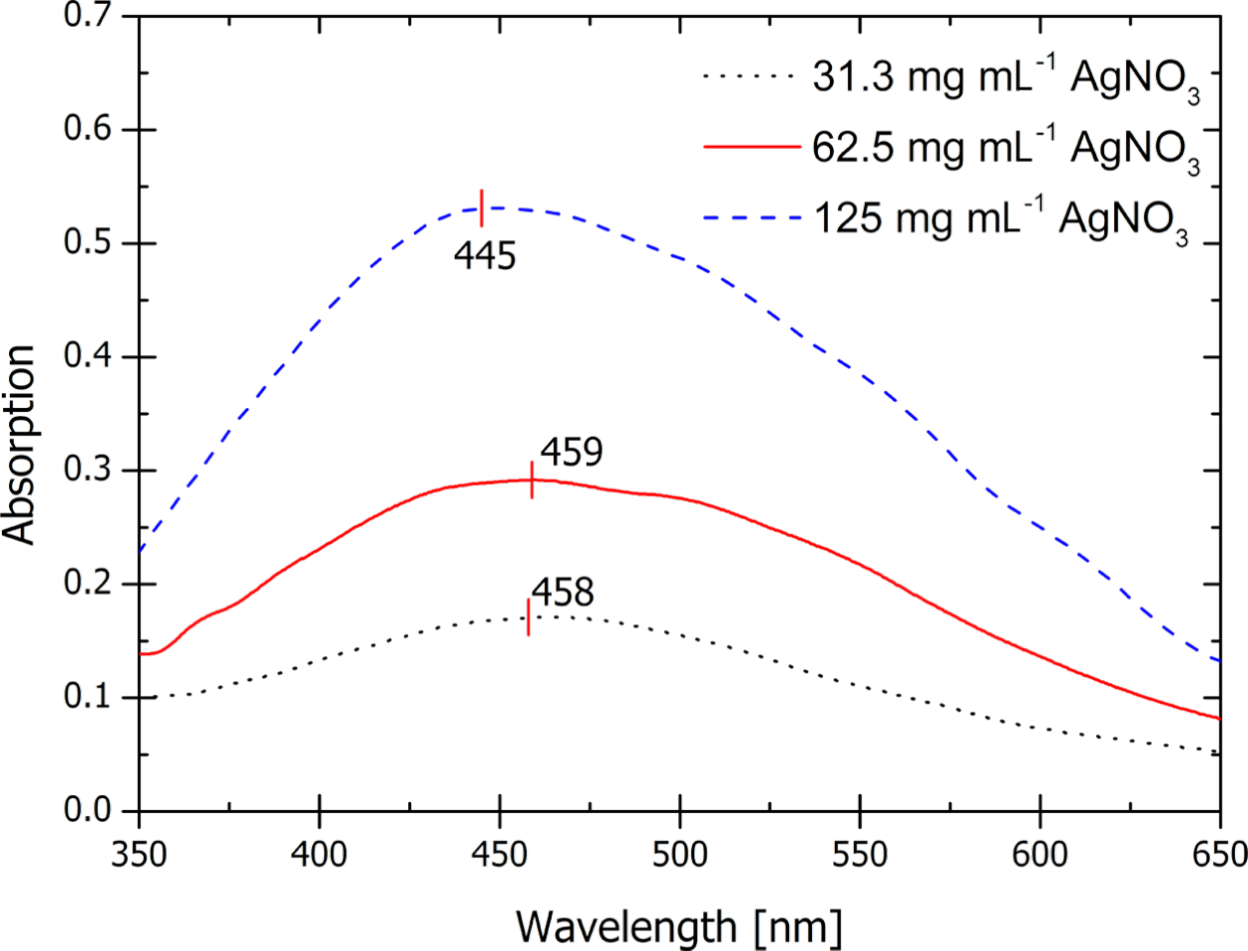
UV–vis absorption spectra of silver nanocomposites at silica wafers after post-exposure bake at 95 °C with varying AgNO_3_ precursor concentrations. The absorption increases with increasing amounts of AgNO_3_ precursor added.

Mostly, AgNPs are formed during the heat treatments before and after UV exposure. The exact process is not known as acetonitrile and the many constituents of SU-8 play a role in the NP formation. The AgNPs formed in the SU-8 polymer matrix show a clear plasmonic absorption [16] in the visible region as seen in Figure 2.

**Figure 3:**
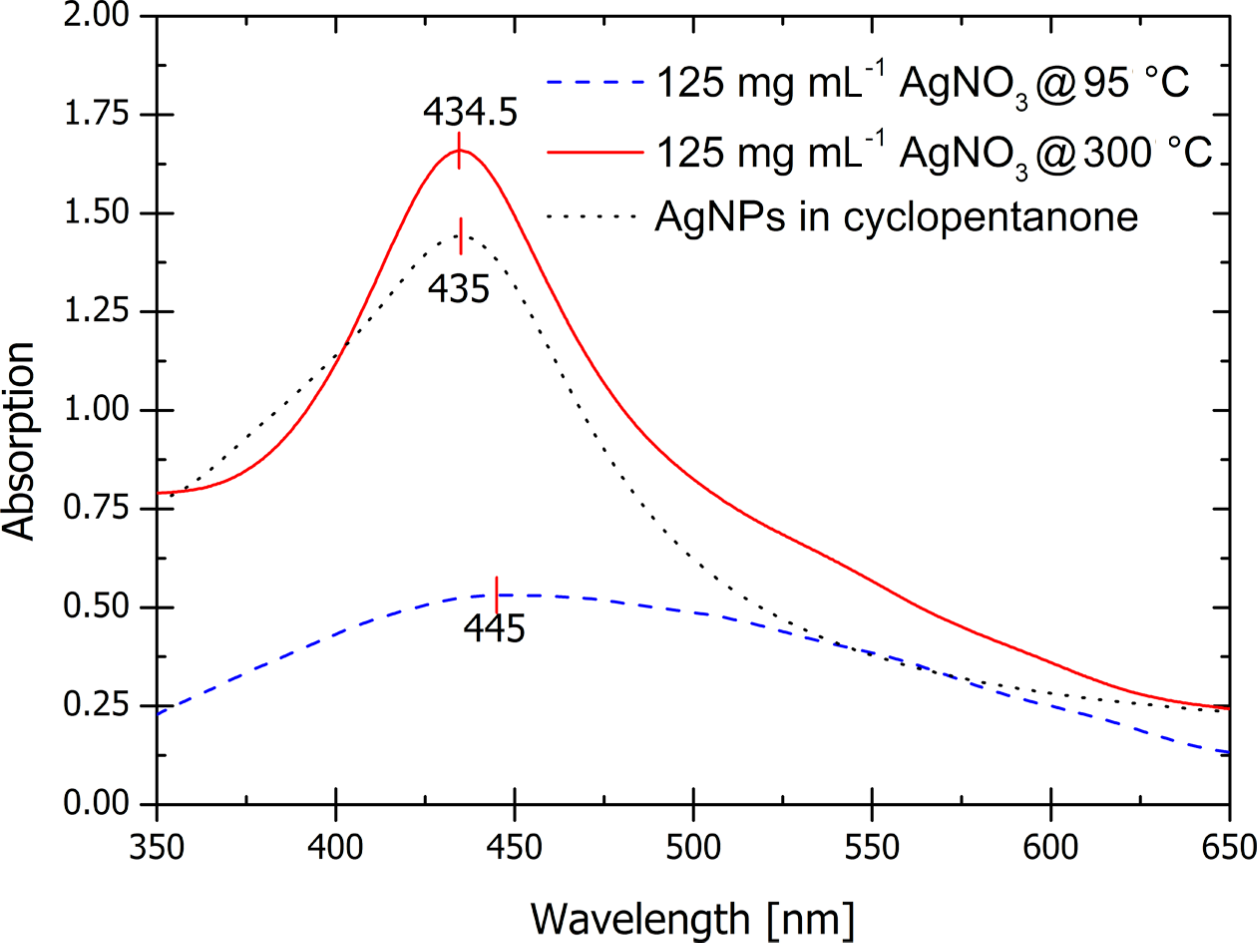
UV–vis absorption spectra of a nanocomposite on a silica wafer containing 0.5 mL of 125 mg·mL^−1^ AgNO_3_ precursor solution after different post-exposure heat treatments. Blue dashed curve – after 95 °C for 10 min, red solid curve – after an additional 300 °C for 30 min and black dotted curve – AgNPs in cyclopentanone for comparison.

The high temperature post-exposure bake at 300 °C resulted in the formation of densely populated silver nanoparticles in the polymer matrix. They appeared to be single nanoparticles entities, smaller than the clusters that formed during the baking steps at 95 °C. This is evident from the UV–vis absorption spectra shown in Figure 3.

**Figure 4:**
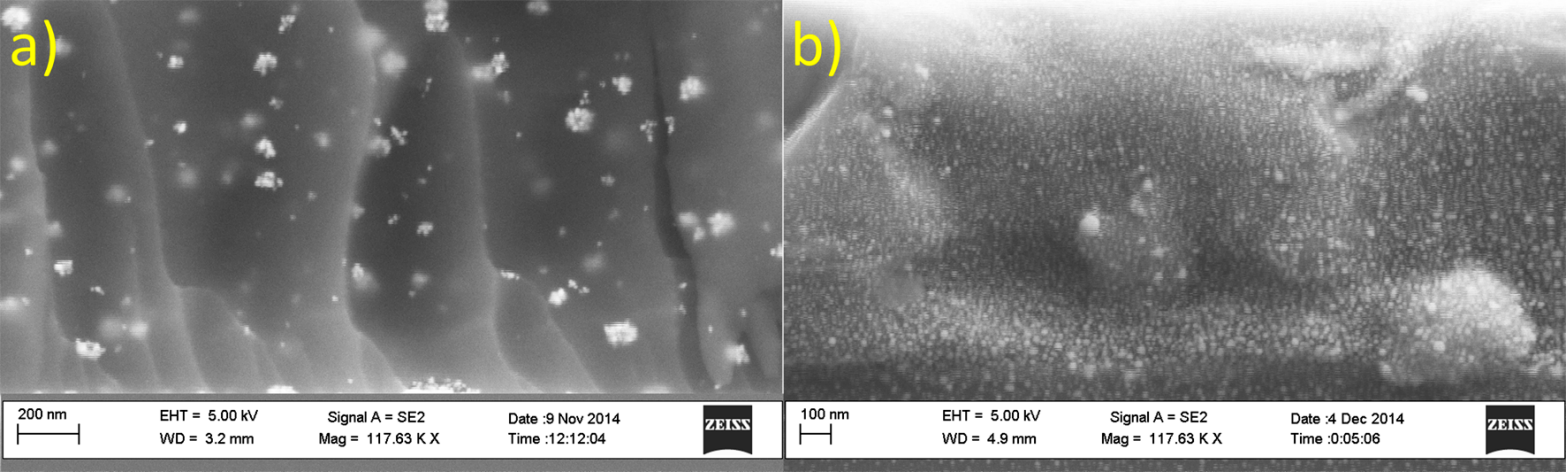
SEM images of a cross section of a nanocomposite on a silicon wafer containing 0.5 mL of 125 mg·mL^−1^ AgNO_3_ precursor solution. a) after a post-bake at 95 °C for 10 min and b) after an additional bake at 300 °C for 30 min.

The plasmonic peak of a nanocomposite baked at 95 °C is broad, which indicates particles or agglomerates with a wide size distribution, whereas the peak corresponding to the composite material treated at 300 °C is sharper, enhanced and more defined with a λ_max_ of 434.5 nm. The peak position at 434.5 nm is a typical value for the absorption band of AgNPs [17]. Also; this peak resembles the plasmonic peak obtained for AgNPs in cyclopentanone. The shoulder appearing at higher wavelengths for the nanocomposite indicates the retention of the AgNP clusters, formed during the lower temperature treatments. These results are visually corroborated in the SEM images shown in Figure 4.

The formation of AgNPs is confirmed with SEM by looking at the cross-sectional area of a fabricated nanocomposite wafer as shown in Figure 4. The images confirm that individual NPs of 25 nm in diameter are formed, although the randomly distributed NP clusters of roughly 80–100 nm are easy to spot when looking at the composite treated at 95 °C. The SEM image further confirms that the additional heat treatment of 300 °C results in the generation of more 25 nm sized nanoparticles. It is important to note that further growth of already formed agglomerated NPs does not happen during this last heat treatment.

**Figure 5:**
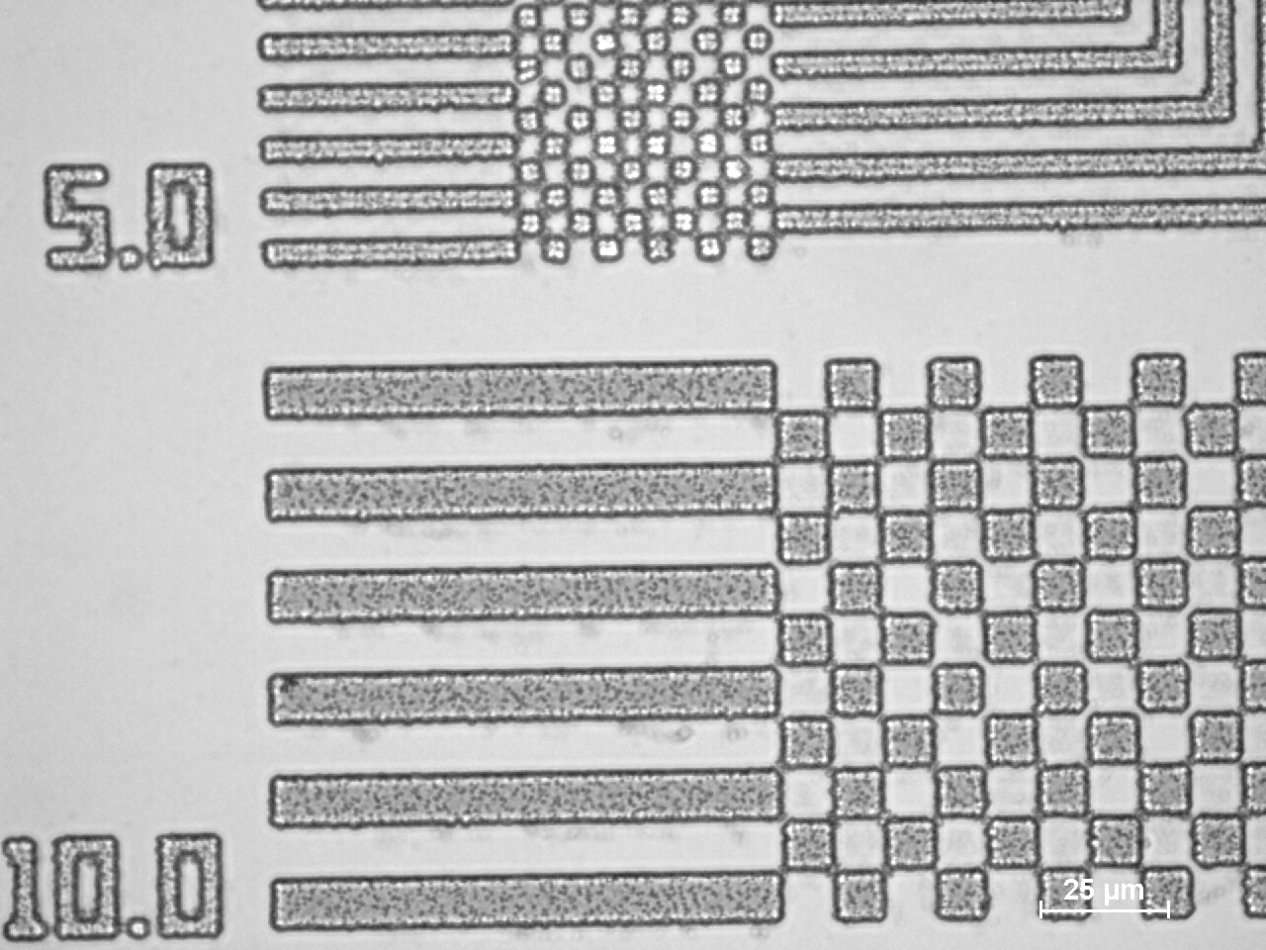
Microscope image at 50× magnification of a nanocomposite containing 0.5 mL of 125 mg·mL^−1^ AgNO_3_ precursor solution after structuring using UV lithography.

Structuring of the nanocomposite is important if to be used in micro- and nanofabrication. Although not fully optimized a resolution of 5 µm is obtained using UV-lithography as shown in Figure 5.

The UV exposure results in the formation of a Lewis acid which cross-links the resist in the exposed areas [18]. The prolonged exposure of 7.5 min compared to a standard exposure time of 10 s is required because of the absorption and shadowing effects of the formed AgNPs. Further optimisation of the exposure time needs to be done for improving the currently obtained resolution of 5 µm. However, preliminary experiments show that the exposure time is independent of the added precursor solution for the structured nanocomposites with concentrations up to 125 mg·mL^−1^ AgNO_3_.

**Figure 6:**
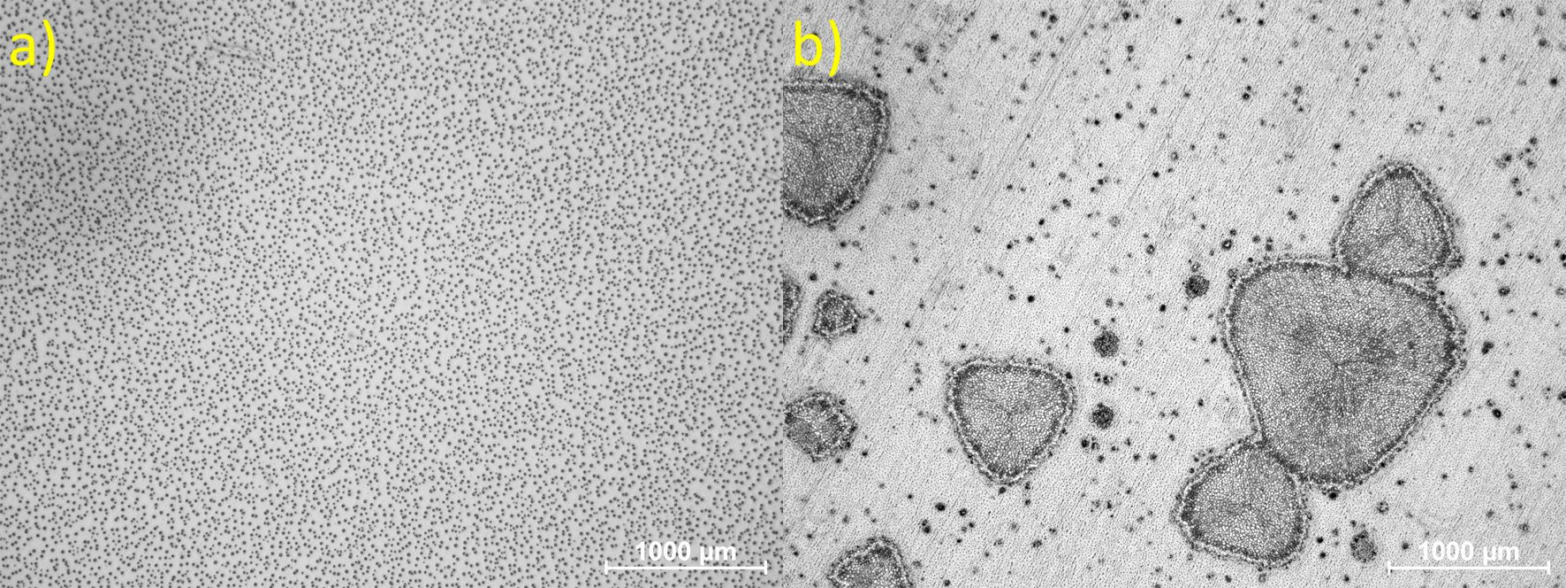
Microscope images at 2.5× magnification of SU-8 nanocomposites after post-exposure bake at 95 °C; a) with 250 mg·mL^−1^ of AgNO_3_ precursor solution added and b) 500 mg·mL^−1^ of AgNO_3_ precursor solution added.

With a concentration of 250 mg·mL^−1^ precursor solution added to the SU-8, larger amounts of homogeneously distributed micron-sized AgNP agglomerates are formed after the post-exposure bake at 95 °C. This is easily seen from the microscope image in Figure 6a. When the precursor solution concentration is increased to 500 mg·mL^−1^, randomly distributed phase separated islands of Ag, more than 100 µm in size, are formed as shown in Figure 6b.

In conclusion, we have developed a method for making in situ SU-8 silver nanocomposites with use of the precursor AgNO_3_ dissolved in the SU-8 compatible solvent acetonitrile. The nanocomposite can easily be deposited and structured by using standard micro- and nanofabrication processes such as spin coating and UV lithography. A high resolution of 5 µm has been achieved with UV lithography. The UV exposure time is found to be independent of the AgNO_3_ precursor concentration. We have shown that a bake at 300 °C results in further AgNP formation in the composite and not particle growth or agglomeration. The plasmonic absorption maximum is close to 435 nm and is independent of the AgNO_3_ precursor concentration up to 125 mg·mL^−1^. The AgNPs formed in the SU-8 matrix is approximately 25 nm and distributed evenly in the composite matrix. At higher precursor concentrations, larger agglomerated NPs are dominant and large islands of phase separated Ag are formed in the composite.

## Experimental

### Preparation of Ag NPs in cyclopentanone

#### Materials

Silver nitrate (AgNO_3_, ≥99.0%) and sodium borohydride (NaBH_4_, ≥98.0%) was bought from Sigma-Aldrich. Luviskol^®^ VA 64, a poly(vinylpyrrolidone-*co*-vinyl acetate) (PVP/VA) mixture was kindly gifted by BASF.

#### Method

1 g of PVP/VA and 0.1 g of silver nitrate is dissolved in 50 mL of absolute ethanol. 0.02 g of sodium borohydride and 0.2 g of PVP/VA dissolved in 10 mL of absolute ethanol is added to the solution at one drop per second under vigorously stirring. 30 s after complete addition the stirring is stopped.

#### Solvent exchange

The solvent exchange is done using a rotary evaporator Rotavapor^®^ R-210 from Büchi.

1. 10 mL of the nanoparticle solution is attached to the rotary evaporator and 1 mL of ethanol evaporated.
2. 1 mL of cyclopentanone is added to the solution.
3. 3 mL of ethanol is evaporated and 1 mL of cyclopentanone added.
4. The remaining 6 mL of ethanol is evaporated and 1 mL cyclopentanone added.
